# Homelessness, justice involvement, and publicly funded substance use treatment after Medicaid expansion

**DOI:** 10.1093/haschl/qxag069

**Published:** 2026-03-24

**Authors:** Suparna Das, Kerry Green

**Affiliations:** Department of Behavioral and Community Health, University of Maryland School of Public Health, College Park, MD 20742, United States; Department of Behavioral and Community Health, University of Maryland School of Public Health, College Park, MD 20742, United States

**Keywords:** Medicaid expansion, homelessness, criminal-justice system, substance use disorder treatment, SUPTRS block grant, public behavioral health systems

## Abstract

**Introduction:**

People experiencing homelessness (PEH) and individuals referred through the criminal-justice (CJ) system face high rates of substance use disorders (SUD) and persistent barriers to treatment. Although Medicaid expansion increased insurance coverage among low-income adults with SUD, it remains unclear how these reforms affected entry into publicly funded treatment for individuals experiencing both homelessness and justice involvement.

**Methods:**

We analyzed 32.9 million specialty SUD treatment admissions reported to the Treatment Episode Data Set–Admissions (TEDS) from 2006 to 2023. Multinomial logistic regression estimated demographic, socioeconomic, clinical, and insurance correlates across pathways. Comparative interrupted time series (CITS) models with state and year fixed effects assessed changes in admissions involving PEH–CJ following Medicaid expansion.

**Results:**

Of all admissions, PEH–CJ admissions showed the highest levels of socioeconomic instability, repeated treatment episodes, and stimulant involvement. Multinomial models identified elevated relative risk among American Indian/Alaska Native, Black, unemployed individuals, those with low educational attainment, and prior treatment episodes. CITS analyses showed no significant post-expansion change in PEH–CJ admission probability.

**Conclusion:**

Medicaid expansion did not alter reliance on publicly funded safety-net treatment for PEH-CJ. Continued dependence on the SUPTRS-BG highlights the need to align Medicaid reforms with strengthened safety-net financing and cross-sector support.

## Introduction

People experiencing homelessness (PEH) are disproportionately affected by the substance-use and overdose crisis, with markedly higher rates of fatal and nonfatal overdose than their housed counterparts^1-3^. PEH also experiences a high prevalence of substance use disorders (SUD) and frequently encounters fragmented access to treatment, often cycling through courts, probation and parole systems, and jails and prisons^4-6^. At the same time, survey estimates indicate that nearly 48-49 million people aged 12 years or older meet criteria for a past-year SUD, underscoring the magnitude of unmet treatment need in the United States.^7^ Within this broader crisis, homelessness and criminal-justice involvement often co-occur, producing distinct patterns of vulnerability that have been insufficiently addressed in policy and treatment system responses^3,8-10^.

Over the past decade, Medicaid reforms have substantially reshaped the behavioral health landscape. The Affordable Care Act (ACA) Medicaid expansion markedly increased insurance coverage among low-income adults with SUD. Building on this expansion, many states have used Section 1115 demonstration waivers to modify Medicaid payment and benefit rules for SUD treatment, including limited waivers of the long-standing “Institutions for Mental Diseases” (IMD) exclusion that historically prohibited federal reimbursement for residential treatment. More recent initiatives, including the 2023 Medicaid Reentry waiver authorizing up to 90 days of pre-release coverage, further extend Medicaid's reach to justice-involved populations. Together, these changes may have altered who enters specialty SUD treatment and how treatment episodes are financed—particularly among individuals facing the highest levels of social and structural risk, such as those experiencing homelessness or referred to through the criminal-justice system. However, Medicaid expansion has not eliminated coverage gaps, especially for individuals with unstable housing or intermittent justice involvement, making it essential to understand how the Substance Use Prevention, Treatment, and Recovery Services Block Grant (SUPTRS-BG) continues to fill remaining gaps in access to publicly funded care.

Despite these major system changes, little is known about how the intersection of homelessness, criminal-justice referral, and Medicaid financing has evolved within the publicly funded specialty treatment system.^10^ Previous research using TEDS described trends in treatment admissions to examine demographic differences across groups.^10^ The present study extends this literature by evaluating how these pathways into treatment changed following Medicaid expansion and by situating treatment entry within the financing context of the publicly funded substance use treatment system, including the SUPTRS-BG.^10^

This study characterizes how homelessness and criminal-justice referral intersect within publicly funded substance use disorder treatment admissions from 2006 to 2023. We assess demographic, socioeconomic, and clinical correlates of 4 mutually exclusive housing–justice referral pathways, with particular attention to admissions involving individuals experiencing homelessness and referred by criminal-justice entities. We further evaluate whether admissions involving this highest-risk group changed following Medicaid expansion, both overall and across race, sex, and age groups. By examining these patterns, this study clarifies the extent to which Medicaid reforms have altered treatment-system contact among populations facing the greatest structural vulnerability and informs understanding of the continuing role of publicly funded safety-net treatment systems in supporting access to care.

## Data source

This study used data from the Treatment Episode Data Set–Admissions (TEDS), a national administrative dataset containing episode-level records of individuals entering publicly funded specialty substance use treatment. TEDS captures standardized demographic, socioeconomic, clinical, and legal characteristics for individuals aged 12 years and older admitted to treatment programs funded or licensed by Single State Agencies (SSAs). The system was established under the Comprehensive Alcohol Abuse, Drug Abuse, and Mental Health Amendments of 1988 (P.L. 100-690) to support federal oversight of services funded through the SUPTRS-BG. The 2 analytic approaches addressed distinct questions. The first analysis characterized the demographic, socioeconomic, and clinical correlates of the 4 housing–justice referral pathways at admission. The second analysis evaluated whether the probability of admissions classified as both experiencing homelessness and criminal-justice referred changed following Medicaid expansion in expansion states relative to non-expansion states, overall and across race, sex, and age groups. This policy analysis estimated differential post-expansion changes between expansion and non-expansion states using state and year fixed effects, rather than relying on a simple comparison of raw pre- and post-expansion means.

### Study population

The analytic sample included treatment admissions reported to TEDS from 2006 through 2023 by all 50 states, the District of Columbia, and Puerto Rico. Each record represents a treatment admission rather than a unique individual. Admissions with complete information on housing status, criminal justice referral, and key demographic and clinical variables were included. The analytic sample included admissions reported between 2006 and 2023, a period that includes the COVID-19 pandemic; no separate adjustment for pandemic-related disruptions was undertaken.

### Outcome measures

The primary outcome was a 4-category measure capturing the intersection of homelessness and criminal justice referral at admission:

1. Not experiencing homelessness and not criminal justice referred (reference)
2. Not experiencing homelessness but criminal justice referred
3. Experiencing homelessness but not criminal justice referred
4. Experiencing homelessness and criminal justice referred

These categories were derived from TEDS variables indicating living arrangement at admission and criminal justice referral source.

### Statistical analysis

Descriptive statistics were used to characterize trends in treatment admissions across outcome categories and demographic, socioeconomic, insurance, and clinical characteristics.

Multinomial logistic regression models were estimated to examine factors associated with each treatment entry pathway relative to the reference group not experiencing homelessness and not criminal justice referred. Covariates included demographic characteristics, socioeconomic factors, treatment history, clinical indicators, and primary substance category.

To examine the relationship between Medicaid expansion and admissions involving individuals experiencing both homelessness and criminal justice referral, we applied a comparative interrupted time series (CITS) design comparing expansion and non-expansion states to estimate changes in the probability of PEH–CJ admissions within publicly funded treatment systems over time. Models were estimated with robust standard errors clustered at the state level, consistent with standard comparative interrupted time series approaches for evaluating policy changes in observational data.^11^ Models included state and year fixed effects, and standard errors were clustered at the state level. Medicaid expansion exposure was coded using state-specific implementation timing, with the first full calendar year of expansion treated as post-expansion for each adopting state.

Additional details on variable construction, regression specification, and missing data handling are provided in the Supplement Methodology. Table S1 lists each state's Medicaid expansion status and the first full calendar year coded as post-expansion in the analysis. This state-specific coding was used to reflect staggered adoption and to classify late-expanding states according to the first full year in which expansion was in effect.

## Results

### Descriptive statistics

Table 1 summarizes 32.9 million publicly funded treatment admissions from 2006 to 2023. Two-thirds (66.9%) involved individuals who were neither experiencing homelessness nor referred by the criminal justice (CJ) system. Another 19.7% were CJ-referred without homelessness, 11.8% involved homelessness without CJ referral, and 1.7% involved both homelessness and CJ referral. White individuals accounted for the largest share of admissions overall, while Black individuals were disproportionately represented in homelessness-related admissions. Adults aged 25-44 years represented the largest age group, with homelessness-related admissions skewing somewhat older. Men accounted for roughly two-thirds of all admissions and more than 70% of CJ-referred admissions. Socioeconomic vulnerability was concentrated among homelessness-related groups: unemployment exceeded 45%, and more than 47% were outside the labor force—substantially higher than the overall sample. Educational attainment was also lower, with roughly one-third lacking a high school diploma. Over time, non-homeless, non-CJ admissions increased to more than 70% after 2020, while CJ-only referrals declined from about 26% in the mid-2000s to 12%-13% by 2023. Homelessness-related admissions increased modestly, from about 10%-11% in 2006 to approximately 14% in 2023. Pregnant and injection-drug–use admissions were concentrated in the non-homeless, non-CJ group, although injection drug use was relatively more common among homelessness-related admissions.

**Table 1:** Characteristics of publicly funded substance use treatment admissions by homelessness and criminal-justice referral status, 2006-2023.

Races	Not experiencing homelessness or criminal justice referred.	Not experiencing homelessness, but criminal justice referred	Experiencing homelessness but not criminal justice referred	Experiencing homelessness and criminal justice referred	Total
American Indian and Alaska Native	2.54	2.75	2.82	7.81	2.70
Asian and Pacific Islander	0.63	0.91	0.54	0.96	0.68
Black/African American	18.41	19.43	26.25	18.95	19.55
White	66.04	63.45	56.39	57.24	64.24
Other race	8.93	11.07	12.33	13.04	9.82
Not reported	3.45	2.39	1.67	2.01	3.01
Total	100.00	100.00	100.00	100.00	100.00
**Ages in years**					
12-17 years	5.05	8.70	0.25	0.81	5.13
18-24 years	14.39	20.66	8.33	12.45	14.88
25-34 years	30.86	31.42	26.83	29.47	30.47
35-44 years	23.40	20.97	27.61	26.09	23.46
45-64 years	25.01	17.54	35.96	30.24	24.92
65+ years	1.29	0.71	1.02	0.95	1.14
Total	100.00	100.00	100.00	100.00	100.00
**Ethnicity**					
Hispanic	12.12	17.74	16.05	19.14	13.81
Non-Hispanic	83.03	80.36	82.64	80.10	82.41
Not reported	4.85	1.89	1.31	0.76	3.78
Total	100.00	100.00	100.00	100.00	100.00
**Sex**					
Male	62.35	73.64	72.68	73.18	65.97
Female	37.58	26.33	27.27	26.77	33.97
Not reported	0.08	0.02	0.05	0.05	0.06
Total	100.00	100.00	100.00	100.00	100.00
**Employed**					
Full time	16.15	26.52	3.04	6.92	16.50
Part-time	6.81	9.54	2.55	4.76	6.81
Unemployed	34.48	31.66	45.45	45.54	35.40
Not in labor force	33.66	31.01	47.25	41.97	34.88
Not reported	8.89	1.26	1.72	0.82	6.41
Total	100.00	100.00	100.00	100.00	100.00
**Education**					
Not a high school graduate	27.30	34.47	31.18	33.29	29.27
High school graduate	39.61	43.89	45.58	46.75	41.28
Some college	17.45	15.65	17.46	15.55	17.07
College or PG	5.78	4.16	4.15	3.16	5.22
Not reported	9.86	1.82	1.62	1.25	7.16
Total	100.00	100.00	100.00	100.00	100.00
**Year of admission**					
2006	62.44	25.37	10.74	1.44	100.00
2007	61.26	26.09	11.18	1.47	100.00
2008	61.86	26.01	10.65	1.47	100.00
2009	62.63	25.46	10.35	1.56	100.00
2010	64.75	23.36	10.32	1.57	100.00
2011	65.74	22.00	10.69	1.57	100.00
2012	65.63	21.47	11.22	1.68	100.00
2013	65.21	21.31	11.53	1.95	100.00
2014	65.27	20.39	12.11	2.24	100.00
2015	68.04	17.58	12.30	2.08	100.00
2016	68.52	17.80	11.73	1.96	100.00
2017	69.83	16.66	11.75	1.77	100.00
2018	69.38	16.44	12.39	1.79	100.00
2019	67.58	17.04	13.56	1.82	100.00
2020	71.94	13.54	12.96	1.56	100.00
2021	73.09	13.35	12.11	1.45	100.00
2022	72.23	13.11	13.38	1.28	100.00
2023	72.10	12.36	14.23	1.31	100.00
Total	66.85	19.71	11.78	1.67	100.00
**Pregnant at admission**	70.90	16.18	11.13	1.79	100.00
**Injection drug users**	68.73	13.02	16.48	1.77	100.00

Percentages for race, age, ethnicity, sex, employment, and education are column percentages, calculated using the following denominators: 22 023 818 episodes (not homeless/not CJ-referred), 6 493 549 episodes (not homeless/CJ-referred), 3 881 012 episodes (homeless/not CJ-referred), and 548 754 episodes (homeless/CJ-referred). Percentages for year of admission, pregnancy at admission, and injection-drug use are row percentages, calculated within each reporting year or subgroup to show how those episodes are distributed across the 4 categories. Row percentages for year of admission are used to align with the cross-sectional, episode-based annual reporting structure of TEDS submissions by Single State.

### Multinomial regression

Table 2 compares admissions among individuals experiencing homelessness, criminal justice (CJ) referral, PEH-CJ, with admissions involving neither condition. Clear demographic and socioeconomic differences emerged across the 3 alternative pathways. Racial disparities were evident across all groups. American Indian/Alaska Native clients were more likely to appear in every category, including more than 3 times as likely to have admissions involving both homelessness and CJ referral (3.23; 95% CI = 3.20-3.27) compared with White clients. Black clients were more likely to have homelessness-only admissions (1.42; 95% CI = 1.41-1.42) and modestly more likely to have admissions involving both homelessness and CJ referral (1.21; 95% CI = 1.20-1.22). Asian/Pacific Islander clients were more likely to enter treatment through CJ-only referrals (1.37; 95% CI = 1.35-1.38) and through the combined homelessness and CJ pathway (1.63; 95% CI = 1.59-1.68). Sex differences were concentrated among CJ referrals: males were more likely to have CJ-only admissions (1.87; 95% CI = 1.77-1.98), but male sex was not significantly associated with admissions involving both homelessness and CJ referral. Age patterns differed sharply across pathways, with adults aged 35-64 years far more likely than adolescents to have homelessness-related admissions. Adults aged 45-64 were markedly more likely to have homelessness-only admissions (24.85; 95% CI = 24.35-25.36) and admissions involving both homelessness and CJ referral (11.55; 95% CI = 11.20-11.91). Socioeconomic factors and treatment history strongly differentiated pathways into care. Individuals with more than 3 prior treatment episodes were more likely to have admissions involving both homelessness and CJ referral (3.55; 95% CI = 3.49-3.60). Lower educational attainment and weak labor force attachment were also strongly associated with homelessness-related admissions; individuals not participating in the labor force were substantially more likely to appear in the combined homelessness and CJ pathway (4.65; 95% CI = 4.51-4.80). Insurance and clinical characteristics further distinguished groups. Admissions involving individuals without insurance were more likely across all 3 categories, including the homelessness and CJ pathway (2.09; 95% CI = 2.07-2.10), while Medicaid coverage was associated with a lower likelihood of admissions involving both homelessness and CJ referral (0.92; 95% CI = 0.91-0.93). Stimulant involvement showed the strongest clinical association with the combined pathway (2.60; 95% CI = 2.59-2.62), whereas opioid involvement was associated with a lower likelihood across all pathways (0.41; 95% CI = 0.40-0.41). Together, these findings indicate that admissions involving both homelessness and CJ referral represent a distinct pathway into treatment characterized by repeated treatment episodes, socioeconomic instability, and stimulant involvement.

**Table 2:** Multinomial logistic regression of factors associated with homelessness and criminal-justice referral pathways in publicly funded SUD treatment admissions, 2006-2023.

Variable	Not homeless but CJ referred RRR (95% CI)	Homeless but not CJ referred RRR (95% CI)	Homeless and CJ referred (PEH–CJ) RRR (95% CI)
Race			
American Indian/Alaska Native	1.15 (1.15-1.16)***	1.35 (1.34-1.35)***	3.23 (3.20-3.27)***
Asian/Pacific Islander	1.37 (1.35-1.38)***	1.14 (1.13-1.16)***	1.63 (1.59-1.68)***
Black/African American	1.11 (1.11-1.12)***	1.42 (1.41-1.42)***	1.21 (1.20-1.22)***
White	Reference	Reference	Reference
Other race	1.19 (1.19-1.19)***	1.47 (1.46-1.48)***	1.60 (1.58-1.61)***
Not reported	1.15 (1.14-1.15)***	1.10 (1.09-1.11)***	1.32 (1.29-1.35)***
Sex			
Male	1.87 (1.77-1.98)***	0.89 (0.84-0.93)***	1.09 (0.96-1.24)
Female	1.21 (1.14-1.28)***	0.54 (0.51-0.56)***	0.60 (0.53-0.68)***
Not reported	Reference	Reference	Reference
Ethnicity (Hispanic)	0.92 (0.91-0.92)***	1.03 (1.03-1.04)***	0.97 (0.96-0.98)***
Age			
12-17	Reference	Reference	Reference
18-24	1.01 (1.01-1.02)***	13.71 (13.43-13.99)***	8.16 (7.91-8.42)***
25-34	0.81 (0.81-0.82)***	18.93 (18.55-19.32)***	9.28 (8.99-9.57)***
35-44	0.71 (0.71-0.72)***	22.68 (22.22-23.14)***	10.37 (10.06-10.70)***
45-64	0.60 (0.59-0.60)***	24.85 (24.35-25.36)***	11.55 (11.20-11.91)***
65+	0.58 (0.58-0.59)***	14.05 (13.74-14.38)***	7.54 (7.24-7.85)***
Prior treatment episodes			
None	2.67 (2.66-2.68)***	0.63 (0.63-0.63)***	2.81 (2.77-2.86)***
One	2.49 (2.48-2.51)***	0.58 (0.58-0.58)***	2.64 (2.60-2.68)***
Two	2.58 (2.57-2.59)***	0.67 (0.66-0.67)***	3.01 (2.96-3.06)***
>3	2.09 (2.08-2.10)***	1.01 (1.00-1.01)*	3.55 (3.49-3.60)***
Not reported	Reference	Reference	Reference
Education			
<High school	2.67 (2.65-2.69)***	3.26 (3.22-3.29)***	3.39 (3.30-3.48)***
High school	2.60 (2.58-2.62)***	3.05 (3.02-3.08)***	3.08 (3.00-3.16)***
Some college	2.24 (2.23-2.26)***	2.83 (2.80-2.86)***	2.51 (2.45-2.58)***
College+	1.83 (1.82-1.85)***	2.23 (2.21-2.26)***	1.71 (1.66-1.76)***
Not reported	Reference	Reference	Reference
Employment status			
Full time	3.90 (3.87-3.93)***	0.38 (0.37-0.38)***	1.28 (1.24-1.32)***
Part time	3.24 (3.21-3.27)***	0.82 (0.81-0.83)***	2.17 (2.10-2.25)***
Unemployed	2.23 (2.21-2.25)***	2.52 (2.49-2.54)***	3.71 (3.59-3.83)***
Not in labor force	2.53 (2.51-2.55)***	2.79 (2.76-2.82)***	4.65 (4.51-4.80)***
Not reported	Reference	Reference	Reference
Injection drug use	1.11 (1.11-1.11)***	1.10 (1.10-1.10)***	1.27 (1.26-1.27)***
Pregnant	0.96 (0.95-0.96)***	1.29 (1.27-1.30)***	1.22 (1.19-1.25)***
Medicaid	1.22(1.21-1.22)***	1.15(1.41-1.15)***	0.99 (0.91-0.93)***
No insurance	2.47 (2.47-2.48)***	1.44 (1.43-1.44)***	2.09 (2.07-2.10)***
Types of substances treated at admissions	1.22 (1.21-1.22)***	1.15 (1.14-1.15)***	0.92 (0.91-0.93)***
Opioids	0.45 (0.45-0.45)***	0.87 (0.87-0.87)***	0.41 (0.40-0.41)***
Hallucinogens	1.27 (1.25-1.28)***	1.05 (1.03-1.07)***	1.30 (1.26-1.34)***
Cannabis	1.28 (1.28-1.29)***	0.75 (0.75-0.75)***	0.92 (0.91-0.92)***
Stimulants	1.52 (1.52-1.52)***	1.87 (1.87-1.88)***	2.60 (2.59-2.62)***
Cocaine	0.91 (0.91-0.92)***	1.27 (1.26-1.27)***	0.87 (0.86-0.88)***
Alcohol	1.06 (1.05-1.06)***	1.29 (1.29-1.30)***	0.98 (0.97-0.98)***
CNS depressants	0.78 (0.78-0.78)***	1.03 (1.02-1.03)***	0.58 (0.57-0.59)***
Other drugs	0.76 (0.76-0.77)***	1.28 (1.28-1.29)***	0.94 (0.92-0.95)***

*P < .05; **P < .01; ***P < .001.

Model statistics: Log pseudolikelihood = −26 325 263.

Pseudo *R*^2^ = 0.121.

### Comparative interrupted time series

Table 3 presents comparative interrupted time-series estimates assessing the association between Medicaid expansion and the probability of publicly funded substance use treatment admissions involving individuals experiencing both homelessness and criminal-justice referral (PEH–CJ). There was no overall post-expansion change in PEH–CJ admissions (−0.001; 95% CI, −0.014-0.013), indicating that Medicaid expansion was not significantly associated with a measurable shift in block-grant–funded treatment entry for this population.

**Table 3:** Comparative interrupted time-series estimates of Medicaid expansion on PEH–CJ treatment admissions.

PEH and CJ	Coefficient	Std. err.	[95% CI]	
**Expansion state × post-expansion period**	−0.001	0.007	−0.014	0.013
**Race**				
American Indian and Alaska Native	0.011	0.006	−0.001	0.022
Asian and Pacific Islander	−0.005	0.003	−0.012	0.001
Black/African American	0.001	0.003	−0.006	0.007
White	−0.005	0.003	−0.012	0.002
Other race	−0.006	0.004	−0.013	0.002
Not reported (reference category)				
**Sex**				
Male	0.007	0.003	0.001	0.014
Female	0.003	0.003	−0.003	0.009
Not reported (reference category)				
**Age**				
12-17 years (reference category )				
18-24 years*	0.014	0.006	0.003	0.026
25-34 years*	0.017	0.007	0.003	0.032
35-44 years*	0.020	0.008	0.005	0.036
45-64 years**	0.022	0.008	0.007	0.037
65+ years*	0.015	0.006	0.003	0.028
**Race interaction expansion state × post-expansion period**				
American Indian and Alaska Native	0.016	0.015	−0.013	0.046
Asian and Pacific Islander	0.006	0.004	−0.002	0.013
Black/African American	0.003	0.004	−0.005	0.011
White	0.004	0.004	−0.003	0.011
Other race	0.006	0.003	−0.001	0.013
Not reported (reference category)				
**Sex interaction expansion state × post-expansion period**				
Male	−0.004	0.003	−0.009	0.002
Female	−0.005	0.003	−0.011	0.000
Not reported (reference category)				
**Age interaction expansion state × post-expansion period**				
12-17 years (reference category)				
18-24 years**	0.005	0.001	0.002	0.007
25-34 years*	0.005	0.002	0.001	0.008
35-44 years	0.003	0.002	−0.001	0.007
45-64 years	0.002	0.002	−0.002	0.006
65+ years	0.002	0.003	−0.004	0.008

*P < .05; **P < .01; ***P < .001.

Estimates are from a comparative interrupted time series (CITS) model with state and year fixed effects. Standard errors are robust to heteroskedasticity and clustered at the state level. The outcome is a binary indicator for admissions involving individuals experiencing both homelessness and criminal-justice referral (PEH–CJ). Model fit statistics include *R*^2^ = 0.0314, adjusted *R*^2^ = 0.0314, within *R*^2^ = 0.0196, and root MSE = 0.1259. The joint *F*-test indicates overall model significance (Prob > *F* < 0.001).

Baseline demographic differences were evident. Males had a higher probability of PEH–CJ admission compared with the reference group (0.007; 95% CI, 0.001-0.014). Adults aged 18-64 had consistently higher probabilities of PEH–CJ involvement than adolescents aged 12-17, with the largest differences observed among individuals aged 45-64 (0.022; 95% CI, 0.007-0.037). Baseline racial differences were small, and all race coefficients had confidence intervals spanning zero.

Post-expansion interaction terms showed no differential effects by race or sex, with all race-by-expansion and sex-by-expansion estimates close to zero and statistically imprecise. In contrast, age-based heterogeneity was observed. Younger adults aged 18-24 (0.005; 95% CI, 0.002-0.007) and 25-34 (0.005; 95% CI, 0.001-0.008) experienced small but measurable increases in PEH–CJ admissions following Medicaid expansion, whereas no significant post-expansion changes were observed for adults aged 35 years and older.

Overall, these results indicate that Medicaid expansion did not reduce reliance on publicly funded treatment for individuals experiencing homelessness and criminal-justice involvement. Block-grant–supported services remained stable following expansion, with only modest increases observed among younger adults.

**Figure 1:**
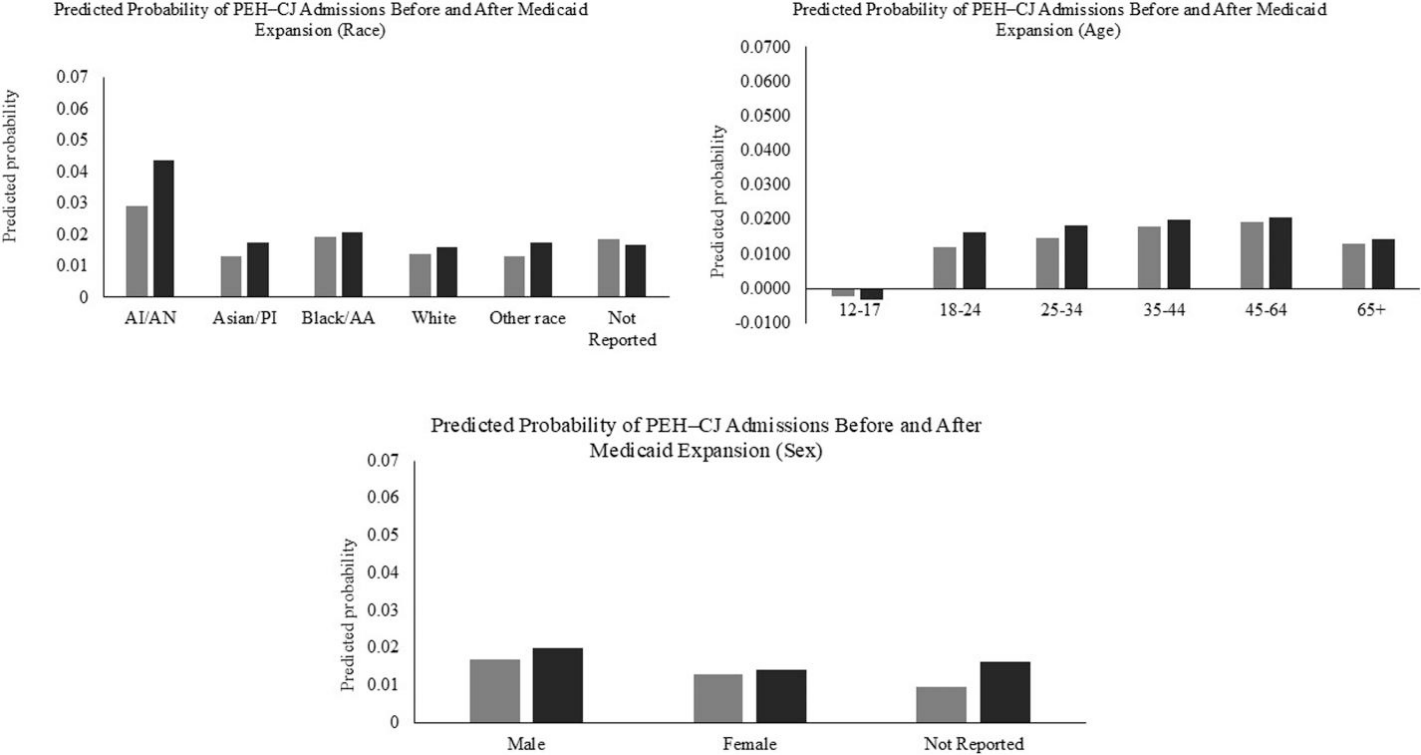
Predicted probability of admissions involving both homelessness and criminal justice referral before and after Medicaid expansion. Predicted probabilities derived from comparative interrupted time series (CITS) models comparing Medicaid expansion and non-expansion states. Light gray bars represent predicted probabilities before Medicaid expansion, and dark bars represent predicted probabilities after expansion. Bars are shown for selected demographic subgroups. PEH–CJ denotes admissions involving both homelessness and criminal justice referral. Source: Treatment Episode Data Set–Admissions (TEDS), 2006-2023.

Figure 1 shows adjusted predicted probabilities of PEH–CJ treatment admissions before and after Medicaid expansion by race, age, and sex. Across racial and sex groups, predicted probabilities change only modestly and in a largely parallel fashion, indicating no meaningful differential effects of Medicaid expansion. American Indian/Alaska Native individuals consistently exhibit the highest predicted probabilities both before and after expansion. Age-specific patterns show small post-expansion increases concentrated among younger adults (18-34), while adolescents and older adults remain largely stable. Overall, the figure reinforces the main findings that Medicaid expansion did not substantially alter reliance on publicly funded treatment for PEH–CJ populations.

In a placebo/falsification test using a pre-expansion cutpoint, the interaction term was not statistically significant (*β* = 0.0014, 95% CI: −0.0063-0.0090), providing no evidence of a differential pre-policy shift between expansion and non-expansion states (Table S2). Figure S1 shows that PEH-CJ admissions rose modestly over the pre-expansion period in both expansion and non-expansion states, although the trajectories were not identical (Figure S1). Among PEH-CJ admissions, expansion states showed a higher proportion of outpatient admissions and a lower proportion of detoxification admissions in the post-expansion period, whereas non-expansion states remained concentrated in detoxification settings across both periods (Table S3).

## Discussion

Using more than 32 million admissions reported through publicly funded specialty treatment systems from 2006 to 2023, this study provides the most comprehensive national assessment to date of how homelessness, criminal-justice involvement, and Medicaid expansion intersect within substance-use treatment systems.

First, entry into publicly funded treatment remains highly stratified by housing instability, justice involvement, and socioeconomic vulnerability. Although two-thirds of admissions involved individuals who were neither experiencing homelessness nor criminal-justice referred, nearly one-third reflected treatment pathways shaped by homelessness, criminal-justice involvement, or both. The subgroup experiencing both homelessness and criminal-justice referral (PEH–CJ)—while numerically smallest—was the most clinically and socially vulnerable, with high unemployment, low educational attainment, repeated treatment episodes, and disproportionately stimulant-involved admissions.

Second, multinomial regression results demonstrate that PEH–CJ admissions represent a distinct pathway into treatment shaped by persistent structural disadvantage. American Indian/Alaska Native, Black, and other race groups were more likely to experience homelessness-associated admissions, reinforcing longstanding evidence that inequities in housing, policing, and service access shape pathways into publicly funded specialty treatment. Racial patterning differed by pathway, with American Indian/Alaska Native individuals showing the strongest association with combined homelessness and justice involvement. Stimulant use emerged as a key clinical marker of PEH–CJ involvement, in contrast to opioid involvement, which was less common in this pathway. Employment status, education, and prior treatment history sharply differentiated pathways: unemployment and detachment from the labor force were among the strongest predictors of PEH–CJ involvement, and individuals with multiple prior treatment episodes were substantially more likely to enter treatment through this pathway. Admissions involving criminal-justice referral without homelessness, by contrast, showed weaker associations with socioeconomic marginalization, underscoring that justice involvement alone does not capture the depth of vulnerability associated with housing instability. Third, comparative interrupted time series analysis indicates that Medicaid expansion was not associated with a measurable change in the probability of PEH–CJ admissions within publicly funded treatment systems. Predicted probabilities before and after expansion were largely parallel across racial and sex groups, and race-by-expansion interaction estimates were small and imprecise, indicating no meaningful differential racial effects of expansion on PEH–CJ admissions. American Indian/Alaska Native individuals continued to exhibit the highest predicted probabilities both before and after expansion, highlighting the persistence of racial inequities in treatment entry. Modest post-expansion increases were observed among younger adults, particularly those aged 18-34, while adolescents and older adults showed little change; these shifts were small in absolute magnitude and did not alter overall patterns of access. Taken together, these findings indicate that while Medicaid expansion substantially reshaped behavioral health financing nationally, it did not substantially change the concentration of PEH–CJ admissions within publicly funded specialty treatment systems reported through TEDS.

The absence of an aggregate expansion effect—and the stability of predicted probabilities across most subgroups—suggests that Medicaid coverage gains alone did not translate into system-wide changes in how the most structurally vulnerable populations access specialty substance-use treatment.

While Medicaid expansion may have facilitated treatment entry for some newly eligible adults, it did not reverse deeply entrenched disparities or supplant the role of publicly funded specialty treatment programs that operate within state-administered treatment systems. Publicly funded specialty treatment systems reported through TEDS continue to function as a critical safety net for individuals facing housing instability, justice involvement, and interruptions in insurance coverage—populations that Medicaid alone does not consistently reach. These findings build on prior analyses using the TEDS framework^10^ examined treatment admissions across homelessness and criminal-justice involvement categories through 2018 and documented important demographic and substance-use differences across these groups. Extending that work through 2023, our analysis further evaluates how these pathways into treatment evolved during the Medicaid expansion era and situates them within the financing structure of publicly funded specialty treatment systems.

## Policy implications

Although Medicaid spending on behavioral health has expanded substantially since the Affordable Care Act, funding for the SUPTRS-BG has grown comparatively little. SAMHSA Congressional Justifications show the block grant at approximately $1.78 billion in FY2010 and $1.91 billion in FY2024,^12,13^ a modest increase relative to the scale of Medicaid's post-expansion growth. Against this backdrop, our findings indicate that individuals experiencing homelessness and those with criminal-justice involvement remained concentrated within the publicly funded specialty treatment systems reported through TEDS, reflecting persistent insurance instability, administrative barriers, and broader structural obstacles that Medicaid coverage alone has not resolved.

The absence of a measurable decline in admissions involving individuals experiencing both homelessness and criminal-justice referral following Medicaid expansion suggests that expanded coverage did not substantially alter treatment-system contact for the most structurally vulnerable populations within these publicly funded systems. Rather than indicating direct financing by the block grant for individual episodes, these findings are consistent with the continuing importance of the state-administered specialty treatment infrastructure monitored through TEDS, including services supported through SUPTRS-BG. In that sense, Medicaid expansion and block-grant-supported treatment infrastructure appear to play complementary rather than fully substitutable roles within the substance use treatment system.

Taken together, these findings highlight the need to modernize and realign the SUPTRS-BG to function effectively alongside Medicaid. This effort should include reevaluating the National Outcome Measures (NOMs), which underpin SUPTRS-BG performance reporting, aligning performance metrics with Medicaid's evolving substance-use disorder benefit structure, and right-sizing state reporting requirements to reduce administrative burden while improving data interoperability. Aligning these measures with modern health data infrastructure could reduce state reporting burden while improving interoperability across federal behavioral health data systems. Strengthening the block grant in these ways would preserve its role as a flexible payer of last resort for individuals who are uninsured, intermittently insured, or face administrative barriers to continuous Medicaid coverage.

Federal policy could further support coordination across financing streams. The Department of Health and Human Services could clarify how SUPTRS-BG resources may complement Medicaid in supporting care coordination, case management, and housing-linked substance use services for individuals experiencing homelessness or justice involvement. Enabling states to braid—rather than silo—funding could promote more continuous engagement even if Medicaid eligibility fluctuates. Greater alignment between CMS and SAMHSA planning processes could also reduce duplicative administrative burden, improve efficiency, and support more seamless care pathways for populations that continue to depend on both Medicaid and publicly funded specialty treatment systems.

### Limitations

First, the analysis relies on the TEDS, which captures treatment admissions to publicly funded programs rather than unique individuals and may under-represent clinical, housing, or social indicators that are inconsistently reported across states. Although states are required to submit these data as a condition of receiving SUPTRS-BG funding, variation in reporting completeness and data system capacity may affect comparability across jurisdictions and over time. Second, TEDS contains limited information on treatment intensity, service quality, and post-admission outcomes, precluding assessment of downstream engagement or clinical improvement. Third, while the multinomial and comparative interrupted time series models adjust for observed demographic and clinical characteristics and include state and year fixed effects, residual confounding from unmeasured state policies, local service capacity, or changes in referral practices cannot be ruled out. Finally, because the analysis is episode-level and observational, repeated admissions for the same individual cannot be linked, and findings should be interpreted as describing system-level patterns rather than individual-level causal effects.

## Conclusion

Homelessness and criminal-justice referral remain major pathways into publicly funded substance use disorder treatment and are closely associated with pronounced socioeconomic and clinical vulnerability. Although Medicaid expansion substantially reshaped behavioral health financing nationally, it was not associated with an overall change in admissions involving individuals experiencing both homelessness and justice involvement. Predicted probabilities remained largely stable across demographic groups, with no evidence of differential post-expansion effects by race or sex and only modest changes among younger adults.

The persistence of these admission patterns within publicly funded specialty treatment systems reported through TEDS—alongside persistent racial disparities—suggests that Medicaid coverage expansion alone has not fundamentally altered treatment-system contact for the most structurally marginalized populations. Strengthening coordination between Medicaid, the SUPTRS-BG safety net, and cross-sector supports such as housing, employment, and reentry services will be essential to addressing the intersecting risks of homelessness, justice involvement, and substance use in the Medicaid-expansion era.

## Supplementary Material

qxag069_Supplementary_Data

## Data Availability

Publicly available data from https://www.samhsa.gov/data/.

## Clinical trial registration number and website

NA.
