# Supplementary material for: Homelessness, justice involvement, and publicly funded substance use treatment after Medicaid expansion
Source: Health Aff Sch. 2026 Mar 24;4(4):qxag069. doi: 10.1093/haschl/qxag069 (PMC13122626; doi:10.1093/haschl/qxag069)
Supplement: qxag069_Supplementary_Data [file qxag069_supplementary_data.zip › Supp Tab 1. State-specific Medicaid expansion.docx]

Supplement Table 1. State-specific Medicaid expansion coding used in the analysis.

| **State or Territory** | **Expansion status in analysis** | **First full calendar year coded as post-expansion** |
| --- | --- | --- |
| Alabama | Not expanded | — |
| Alaska | Expanded | 2016 |
| American Samoa | Not applicable | — |
| Arizona | Expanded | 2014 |
| Arkansas | Expanded | 2014 |
| California | Expanded | 2014 |
| Colorado | Expanded | 2014 |
| Connecticut | Expanded | 2014 |
| Delaware | Expanded | 2014 |
| District of Columbia | Expanded before 2014 | 2014 |
| Federated States of Micronesia | Not applicable | — |
| Florida | Not expanded | — |
| Georgia | Not classified as ACA expansion in analysis | — |
| Guam | Not applicable | — |
| Hawaii | Expanded | 2014 |
| Idaho | Expanded | 2020 |
| Illinois | Expanded | 2014 |
| Indiana | Expanded | 2015 |
| Iowa | Expanded | 2014 |
| Kansas | Not expanded | — |
| Kentucky | Expanded | 2014 |
| Louisiana | Expanded | 2017 |
| Maine | Expanded | 2019 |
| Marshall Islands | Not applicable | — |
| Maryland | Expanded | 2014 |
| Massachusetts | Expanded before 2014 | 2014 |
| Michigan | Expanded | 2015 |
| Minnesota | Expanded before 2014 | 2014 |
| Mississippi | Not expanded | — |
| Missouri | Expanded | 2022 |
| Montana | Expanded | 2016 |
| Nebraska | Expanded | 2021 |
| Nevada | Expanded | 2014 |
| New Hampshire | Expanded | 2015 |
| New Jersey | Expanded | 2014 |
| New Mexico | Expanded | 2014 |
| New York | Expanded before 2014 | 2014 |
| North Carolina | Implemented in 2023; no full post-expansion year in study period | — |
| North Dakota | Expanded | 2014 |
| Northern Mariana Islands | Not applicable | — |
| Ohio | Expanded | 2014 |
| Oklahoma | Expanded | 2022 |
| Oregon | Expanded | 2014 |
| Palau | Not applicable | — |
| Pennsylvania | Expanded | 2015 |
| Puerto Rico | Not applicable | — |
| Rhode Island | Expanded | 2014 |
| South Carolina | Not expanded | — |
| South Dakota | Implemented in 2023; no full post-expansion year in study period | — |
| Tennessee | Not expanded | — |
| Texas | Not expanded | — |
| Utah | Expanded | 2020 |
| Vermont | Expanded before 2014 | 2014 |
| Virgin Islands | Not applicable | — |
| Virginia | Expanded | 2019 |
| Washington | Expanded | 2014 |
| West Virginia | Expanded | 2014 |
| Wisconsin | Partial coverage; not classified as ACA expansion in analysis | — |
| Wyoming | Not expanded | — |

**Notes:** Expansion timing was coded using the first full calendar year in which Medicaid expansion was in effect in each state. States that implemented expansion in 2023 did not contribute post-expansion observations within the 2006–2023 study period. Jurisdictions with expansion in place before 2014 were coded as expanded beginning in 2014 for consistency with the analytic framework. States with partial or waiver-based approaches that did not constitute full ACA Medicaid expansion were not classified as expansion states in the main analysis.
