# Supplementary material for: Homelessness, justice involvement, and publicly funded substance use treatment after Medicaid expansion
Source: Health Aff Sch. 2026 Mar 24;4(4):qxag069. doi: 10.1093/haschl/qxag069 (PMC13122626; doi:10.1093/haschl/qxag069)
Supplement: qxag069_Supplementary_Data [file qxag069_supplementary_data.zip › Supp Tab 2. Falsification Test Pre-Expansion Cutpoint.docx]

Supplement Table 2: Placebo/Falsification Test Using a Pre-Expansion Cutpoint

| Placebo interaction (Post-2011 × expansion state) | Placebo year | Estimate | 95% CI |
| --- | --- | --- | --- |
| PEH + CJ admission share | 2011 | 0.0014 | -0.0063, 0.0090 |

Estimates are from a linear probability model using collapsed state-year data for the pre-expansion period only, with state and year fixed effects and standard errors clustered at the state level. Outcome is the annual share of admissions classified as PEH + criminal-justice referred. Model statistics: R-squared = 0.6777; adjusted R-squared = 0.6223; within R-squared = 0.0154.
