# Supplementary material for: Homelessness, justice involvement, and publicly funded substance use treatment after Medicaid expansion
Source: Health Aff Sch. 2026 Mar 24;4(4):qxag069. doi: 10.1093/haschl/qxag069 (PMC13122626; doi:10.1093/haschl/qxag069)
Supplement: qxag069_Supplementary_Data [file qxag069_supplementary_data.zip › Supp Tab 3. Distribution of broad service setting.docx]

Supplement Table 3. Distribution of broad service setting among PEH-CJ admissions before and after Medicaid expansion, by expansion status

| Broad service setting | Expansion states, pre | Expansion states, post | Non-expansion states, pre-2014 | Non-expansion states, 2014–2023 | Total |
| --- | --- | --- | --- | --- | --- |
| Detox | 67,573 (28.01) | 48,629 (20.28) | 11,238 (50.66) | 23,629 (51.88) | 151,069 (27.53) |
| Residential | 78,508 (32.55) | 77,584 (32.35) | 6,102 (27.51) | 11,467 (25.18) | 173,661 (31.65) |
| Outpatient | 95,128 (39.44) | 113,602 (47.37) | 4,842 (21.83) | 10,452 (22.95) | 224,024 (40.82) |
| Total | 241,209 (100.00) | 239,815 (100.00) | 22,182 (100.00) | 45,548 (100.00) | 548,754 (100.00) |

Values are number of admissions, with column percentages in parentheses. The analysis was restricted to admissions classified as both people experiencing homelessness and criminal-justice referred (PEH-CJ). Service settings were grouped into detoxification, residential, and outpatient categories. Expansion-state periods were defined using state-specific first full calendar year of Medicaid expansion; non-expansion states were divided into pre-2014 and 2014–2023 comparison periods.
