# Supplementary material for: Homelessness, justice involvement, and publicly funded substance use treatment after Medicaid expansion
Source: Health Aff Sch. 2026 Mar 24;4(4):qxag069. doi: 10.1093/haschl/qxag069 (PMC13122626; doi:10.1093/haschl/qxag069)
Supplement: qxag069_Supplementary_Data [file qxag069_supplementary_data.zip › Supplement Methodology_RR1.docx]

**Supplement Methodology**

**Data Source and Reporting Context**
TEDS is a national administrative reporting system of episode-level records submitted through state substance use treatment reporting systems. TEDS captures standardized information on individuals aged 12 years or older entering specialty substance use treatment, including demographic characteristics, socioeconomic status, substance use patterns, treatment history, and legal involvement. TEDS was established following the Comprehensive Alcohol Abuse, Drug Abuse, and Mental Health Amendments of 1988 (P.L. 100-690), which required federal monitoring of services supported through the Substance Abuse Prevention and Treatment Block Grant, now the Substance Use Prevention, Treatment, and Recovery Services Block Grant (SUPTRS-BG). As such, TEDS serves as a core federal reporting system for monitoring access to and characteristics of publicly funded specialty substance use treatment and informing block-grant oversight. TEDS data are submitted by all 50 states, the District of Columbia, and Puerto Rico through state-administered reporting systems. Although SAMHSA applies annual validation and standardization procedures, differences in state data infrastructure and reporting practices may affect completeness and comparability across jurisdictions.

**Study Population**
Each TEDS record represents a formal admission to a specialty substance use treatment program and excludes prescreening, referral-only encounters, and administrative contacts. Because TEDS is admission-based rather than person-based, repeated treatment episodes for the same individual may appear as separate records. The analytic sample included admissions reported between 2006 and 2023. Records were retained if they contained sufficient information to classify housing status and criminal justice referral at admission; covariates with “not reported” values were retained as separate analytic categories rather than excluded.

**Outcome Construction**
The primary multinomial outcome captured four mutually exclusive admission pathways reflecting the intersection of homelessness and criminal justice referral:

1. Not experiencing homelessness and not criminal justice referred (reference)
2. Not experiencing homelessness but criminal justice referred
3. Experiencing homelessness but not criminal justice referred
4. Experiencing homelessness and criminal justice referred (PEH-CJ)

These categories were constructed using TEDS indicators for living arrangement at admission and criminal justice referral. For the comparative interrupted time series analysis, the outcome was a binary indicator for whether an admission was classified as PEH-CJ.

**Covariates**
Multinomial models included demographic, socioeconomic, and clinical covariates: race, sex, ethnicity, age group, prior treatment episodes, education, employment status, pregnancy at admission, injection drug use, insurance status, and primary substance category. Insurance was represented using indicators for Medicaid coverage and no insurance. Substance categories included opioids, hallucinogens, cannabis, stimulants, cocaine, alcohol, central nervous system depressants, and other drugs. Categories for “not reported” were retained where applicable.

**Multinomial Regression**
We used multinomial logistic regression because the four admission pathways are nominal and non-ordered. Models estimated the relative association of each pathway compared with the reference category of admissions involving neither homelessness nor criminal justice referral. Robust standard errors were used to account for heteroskedasticity in admission-level data.

**Comparative Interrupted Time Series**
To evaluate whether Medicaid expansion was associated with changes in admissions involving both homelessness and criminal justice referral, we implemented a comparative interrupted time series (CITS) design. Expansion states served as the policy-exposed group and non-expansion states as the comparison group. Medicaid expansion exposure was coded using state-specific implementation timing rather than a single national post-2014 indicator; for each adopting state, admissions were classified as post-expansion beginning in the first full calendar year in which expansion was in effect in that state, and admissions in earlier years were classified as pre-expansion. States that did not expand Medicaid during the analytic period contributed only comparison observations. Supplement Table 1 lists each state’s expansion status and the first full calendar year coded as post-expansion.

The primary CITS models were estimated as linear probability models with state and year fixed effects and standard errors clustered at the state level. The main specification interacted the expansion-period indicator with race, sex, and age group to examine whether the association between Medicaid expansion and PEH-CJ admissions varied across subgroups.

$${PEH-CJ}_{st}= \beta_{0}+ \beta_{1} \left( {ExpansionStates}_{s} \times{Post}_{st} \right)+ \gamma_{s}+ \delta_{t}+\varepsilon_{st}$$

where $PEH\text{-}CJ_{st}$indicates whether an admission in state $s$and year $t$was classified as both experiencing homelessness and criminal-justice referred; $ExpansionState_{s}$denotes states that adopted Medicaid expansion during the study period; and $Post_{st}$denotes the state-specific post-expansion period, defined as the first full calendar year in which Medicaid expansion was in effect in that state and all subsequent years. The term $\gamma_{s}$represents state fixed effects, $\delta_{t}$represents year fixed effects, and $\varepsilon_{st}$is the error term. Standard errors were clustered at the state level. In subgroup analyses, the expansion-period term was interacted with race, sex, and age group to assess whether the association between Medicaid expansion and PEH-CJ admissions varied across demographic groups.

Because Medicaid expansion occurred on a staggered basis, this fixed-effects specification should be interpreted as a policy-oriented model in a staggered-adoption setting. As in related difference-in-differences applications, estimates may be sensitive to treatment-effect heterogeneity across expansion cohorts or over time. Accordingly, findings from the main CITS analysis were interpreted cautiously as policy-associated estimates.

**Supplemental Design Checks**
We conducted two supplemental checks to evaluate the CITS design. First, we assessed pre-policy comparability by plotting annual mean state-year shares of admissions classified as PEH-CJ separately for expansion and non-expansion states during the pre-expansion period (2006–2013). Admissions were collapsed to the state-year level and then averaged within expansion groups for each year.

Second, we conducted a placebo/falsification test using a pre-expansion cutpoint (2011). Using the collapsed state-year file restricted to the pre-expansion period, we re-estimated the main fixed-effects model with state and year fixed effects and state-clustered standard errors. This test was intended to assess whether the null main findings could plausibly reflect spurious differential pre-policy shifts between expansion and non-expansion states.

**Missing Data**
TEDS is an administrative reporting system rather than a survey, and missingness primarily reflects variation in state reporting systems rather than respondent nonresponse. Data are submitted through state reporting systems to SAMHSA as part of federal reporting processes tied to oversight of the Substance Use Prevention, Treatment, and Recovery Services Block Grant (SUPTRS-BG). Variables coded as “not reported” were retained and modeled explicitly rather than excluded. No imputation procedures were applied.
