# Supplementary figures and images for: Homelessness, justice involvement, and publicly funded substance use treatment after Medicaid expansion
Source: Health Aff Sch. 2026 Mar 24;4(4):qxag069. doi: 10.1093/haschl/qxag069 (PMC13122626; doi:10.1093/haschl/qxag069)

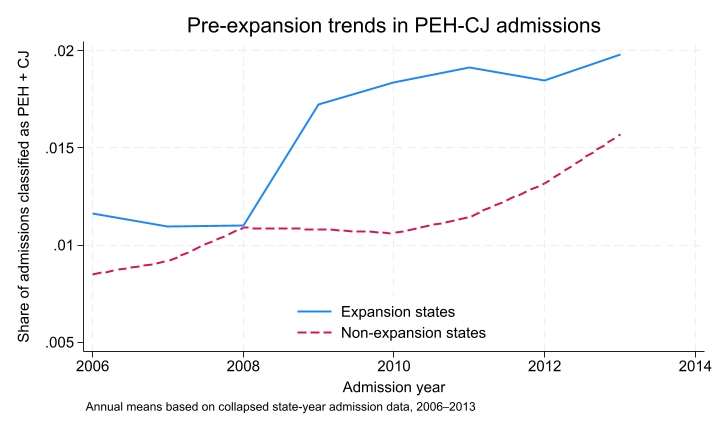

Supplement: qxag069_Supplementary_Data [file qxag069_supplementary_data.zip › Supplement Fig. 1 pre and post admission.jpg]
